# Case report: Mexiletine suppresses ventricular arrhythmias in Andersen-Tawil syndrome

**DOI:** 10.3389/fcvm.2022.992185

**Published:** 2022-08-25

**Authors:** Jing Yang, Kun Li, Tingting Lv, Ying Xie, Fang Liu, Ping Zhang

**Affiliations:** ^1^School of Clinical Medicine, Tsinghua University, Beijing, China; ^2^Department of Cardiology, Beijing Tsinghua Changgung Hospital, School of Clinical Medicine, Tsinghua University, Beijing, China

**Keywords:** mexiletine, ventricular arrhythmias, Andersen-Tawil syndrome, case report, KCNJ2

## Abstract

It is arduous to determine clinical solutions for Andersen-Tawil syndrome (ATS) in patients intolerant of β-blocker. Here, we present the case of a 7-year-old boy with periodic paralysis and dysmorphic features who experienced syncope four times during exercise. His ECG revealed enlarged U waves and QU-prolongation associated with ATS-specific U wave patterns, frequent PVCs, and non-sustained bidirectional or polymorphic ventricular tachycardia. The genetic test showed a *de novo* missense R218W mutation of *KCNJ2*. With the diagnosis of ATS and intolerance of β-blocker, the patient was prescribed oral medications of mexiletine 450 mg/day without severe adverse effects. The repeat ECG showed decreased PVC burden from 38 to 3% and absence of ventricular tachycardia. He remained symptom-free during over 2 years of outpatient follow-up. This case demonstrates a new anti-arrhythmic therapy with mexiletine for prevention of life-threatening cardiac events in patients with ATS who are intolerant of β-blocker treatment.

## Introduction

Andersen-Tawil syndrome (ATS) is a rare hereditary arrhythmia disease also classified as long QT syndrome type 7 (LQT7) and is manifested as ventricular arrhythmias (VAs), periodic paralysis, and dysmorphic features. In patients diagnosed with ATS, β-blockers and/or flecainide is primarily recommended according to a Heart Rhythm Society (HRS) expert consensus statement (1). However, it is arduous to determine clinical solutions for patients intolerant of β-blocker considering the lack of access to flecainide in China. Recently, several studies reported that patients with LQT types 1–3 were responsive to anti-arrhythmic mexiletine. However, the efficacy of mexiletine in patients with ATS (LQT7) with *KCNJ2* mutation was rarely reported. Here, we present the effective treatment of a boy who suffered from ATS and recurrent syncope with oral mexiletine for complex ventricular arrhythmias.

## Case description

A 7-year-old boy was brought to the local hospital with a main complaint of experiencing syncope four times. The first episode happened during a tug-of-war at the kindergarten about 2 years ago before the visit to our hospital; the boy reported palpitation with amaurosis followed by loss of consciousness without limb convulsions, tongue bites, or incontinence. The boy regained consciousness spontaneously after 20 s and experienced palpitations for the following 10 min. The patient experienced three additional episodes during exercising, climbing stairs, and lifting heavy objects. A 24-h ambulatory electrocardiogram (ECG) demonstrated frequent premature ventricular contractions (PVCs) in bigeminy and non-sustained bidirectional ventricular tachycardia (VT). His serum potassium was 3.9 mmol/L, and the echocardiograms revealed normal cardiac structure and function. Both head computer tomography and electroencephalogram (EEG) appeared to be normal. The local doctors then considered the diagnosis of catecholaminergic polymorphic ventricular tachycardia (CPVT) and prescribed metoprolol 11.25 mg twice per day. He manifested apparent fatigue and cold extremities after taking the pill just for 2 days. Successive electrocardiograms showed sinus bradycardia, and he stopped taking the medication. Two months later, the boy lost consciousness repeatedly after climbing stairs. Therefore, he was transferred to our institution, a university-affiliated teaching hospital, for further evaluation and treatment.

**Figure 1:**
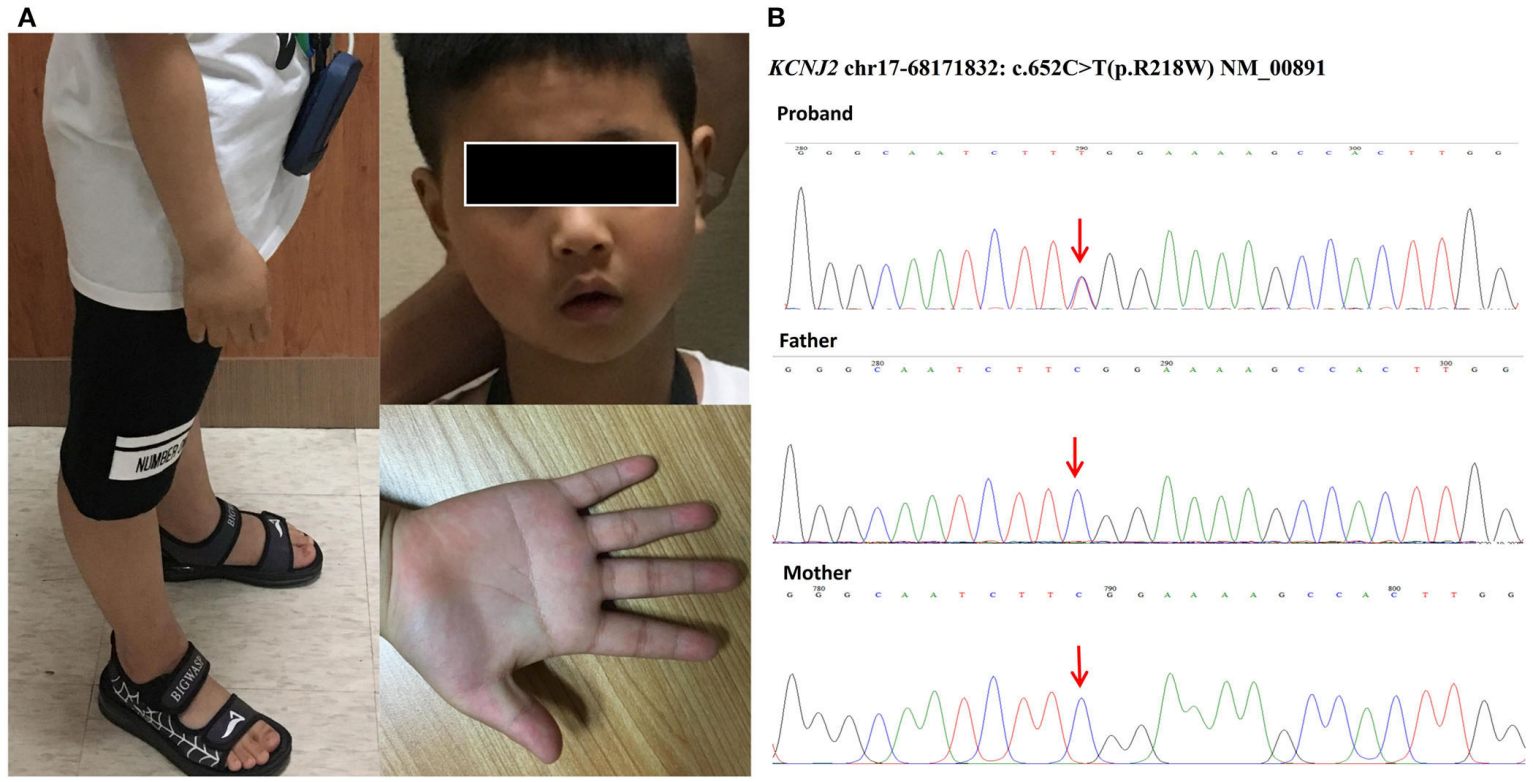
**(A)** Dysmorphology pictures of the boy included mandibular hypoplasia, single palmar crease, and long bone over hyperextension. **(B)** Genetic testing of our patient identified a heterozygous missense mutation named c.652C > T (p.R218W) in the coding region of *KCNJ2*. His parents were tested as wild types without mutations, so our patient carried a *de novo* mutation.

Upon admission, his neurology examination was unremarkable. The physical examination revealed dysmorphology, including mandibular hypoplasia, single palmar crease, and long bone over hyperextension (Figure 1A). His medical history revealed lower limb myasthenia with hypokalemia, but it recovered after potassium supplement. The patient was of normal stature (height of 125 cm) with no family history of sudden cardiac death (SCD).

**Figure 2:**
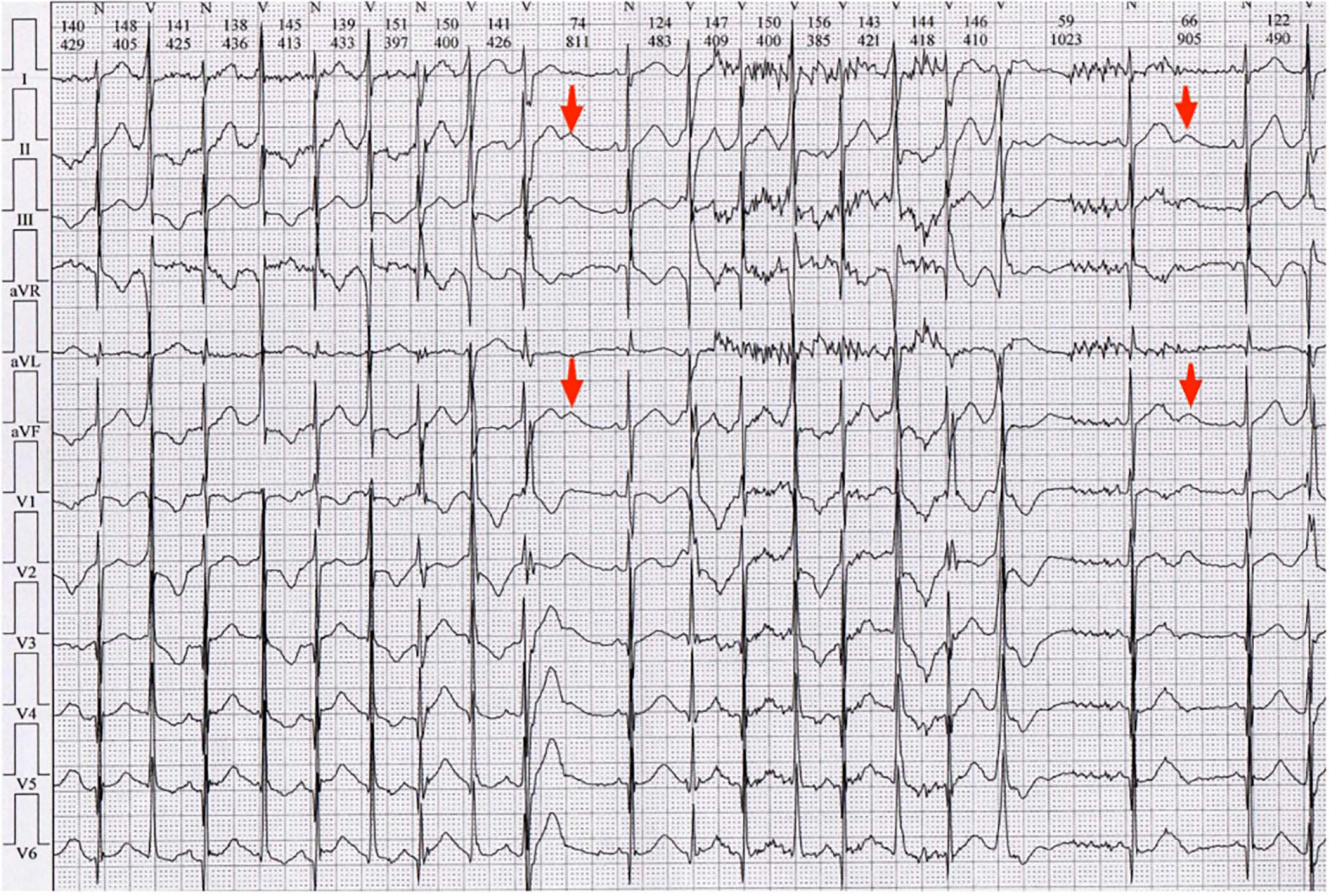
Electrocardiogram (ECG) of the local hospital demonstrated sinus rhythm with frequent PVCs. Enlarged *U* waves (*U* wave is considered enlarged if its amplitude is ≥ 0.15 mV and its duration is ≥ 210 ms, indicated by red arrows) in leads II, III, aVF, V1-V2, and wide T-U junction (Tp-Up, 240 ms) were shown in ECGs. He had QTc of 380 ms and QUc of 671 ms.

The patient's surface electrocardiograms (ECGs) taken in the local hospital demonstrated sinus rhythm with frequent PVCs. Enlarged U waves (U wave is defined as an early diastolic deflection after the end of the T wave and is considered enlarged if its amplitude is ≥ 0.15 mV and its duration is ≥ 210 ms, indicated by red arrows in Figure 2) (2) in leads II, III, aVF, V1-V2, and wide T-U junction (T peak-U peak 240 ms) were shown in ECGs. He had a QTc interval of 380 ms and a QUc interval of 671 ms (QU interval is defined as from onset of QRS to the end of the U wave, and QT and QU intervals are corrected (QTc and QUc) using Bazett's formula for comparison with different heart rates).

A series of electrocardiograms conducted in our hospital revealed frequent PVCs with right bundle branch block (RBBB) morphology and bidirectional PVCs, with QTc of 420 ms and QUc of 680 ms (Supplementary Figure 1). Furthermore, frequent PVCs (38% of total beats) in bigeminy and recurrent asymptomatic non-sustained bidirectional or polymorphic ventricular tachycardia (bVT/pVT, 2,659 times VT) were shown in 24-h Holter recording (GE MARS) (Supplementary Figure 2). Atrial electrical abnormalities were not detected during careful rhythm monitoring. *U* wave was more prominent at a slow heart rate.

**Figure 3:**
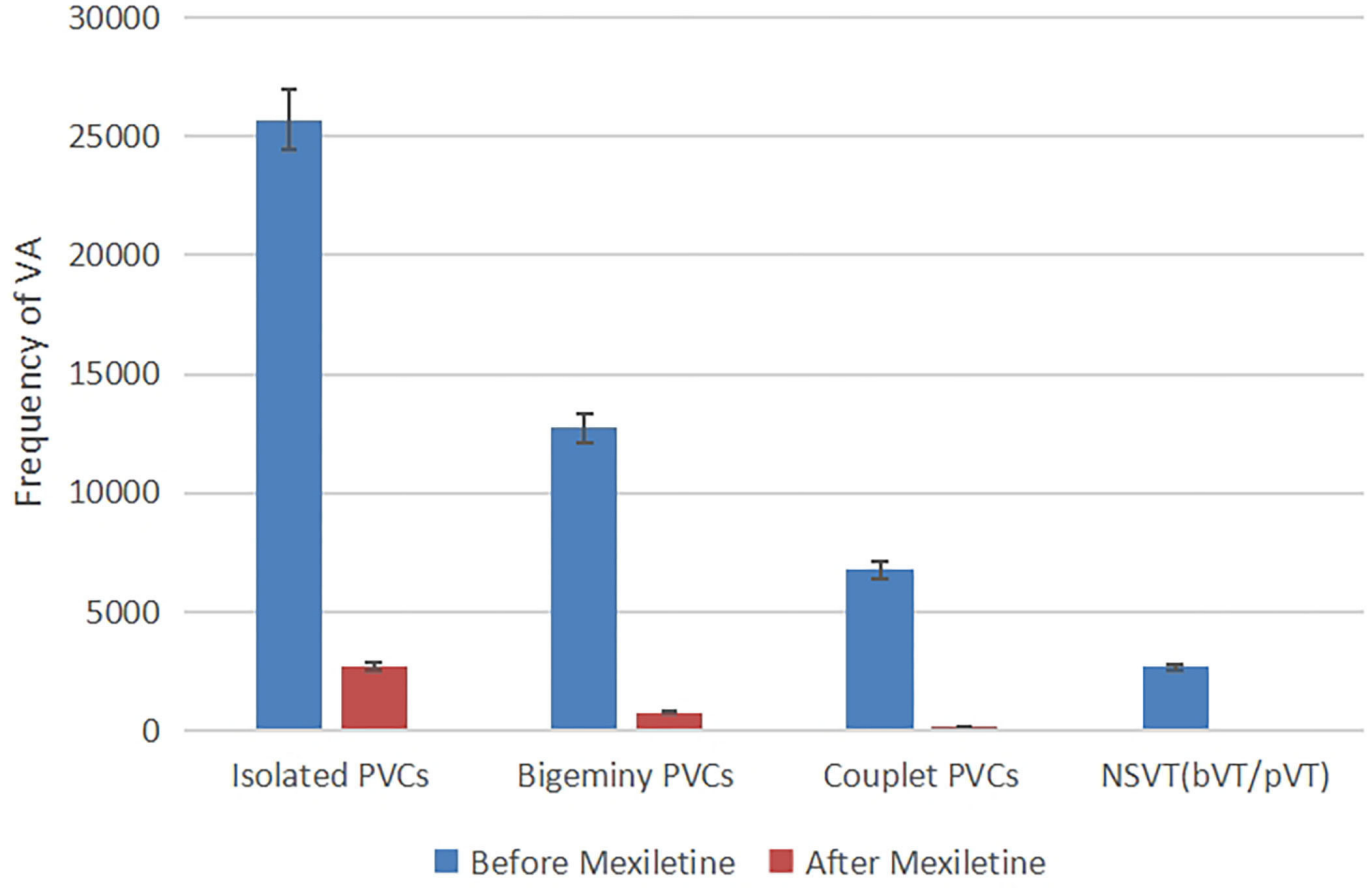
Frequency of ventricular arrhythmia in 24-h Holter recording before and after taking mexiletine 3 days, kinds of ventricular arrhythmias (isolated PVCs, bigeminy PVCs, couplet PVCs, and non-sustained ventricular tachycardia) were suppressed.

The clinical diagnosis of congenital ATS was made based on the typical prolonged QUc interval and enlarged U waves, dysmorphology, and history of paroxysmal hypokalemia. The recurrent syncope was most likely due to sustained VT or ventricular fibrillation (VF). With the diagnosis of ATS and intolerance to β-blocker, the patient was prescribed an oral medication of mexiletine. Considering the high risk of gastrointestinal side effects, a low dose of 300 mg/day (10 mg/kg/day every 8 h) was initially prescribed. Then, the prescribed dosage was gradually increased to 450 mg/day (15 mg/kg/day every 8 h), for which the patient reported occurrence of mild side effects including nausea and loss of appetite. Three days after taking mexiletine, continuous electrocardiograph monitoring showed suppressed ventricular arrhythmias. A 24-h Holter was performed after the treatment showed decreased PVC burden from 38 to 3% of total beats (Figure 3) and absence of ventricular tachycardia. We also measured the patient's electrocardiographic indexes of SCD prediction including heart rate turbulence (turbulence onset and turbulence slope, TO and TS) and T wave alternans, TWA). The result showed a significant difference before and after the treatment (TO 1.84 vs.−1.24%, TS 3.44 vs. 14.17 ms/RR, and TWA 98 uV vs. 77 uV in lead V1). Additionally, the patient was given an exercise stress test (Bruce protocol). The test results revealed a QUc 730 ms at the baseline heart rate 78 bpm and 590 ms during exercise with a heart rate of 121 bpm, a *U* wave infusion with the *P* wave, and a “U on P” sign (*U*-wave masquerading *P*-wave) at peak heart rate 144 bpm (Supplementary Figure 3). Neither ventricular nor atrial arrhythmia was induced during exercise after taking mexiletine.

Considering the genetic characteristics, we screened the ECGs of his 1st-degree blood relatives. The QT interval was completely normal without a U wave in his parents. The genetic testing of our patient identified a heterozygous missense mutation named c.652C > T (p.R218W, NM_00891, Figure 1B) in the C-terminal interaction domain of *KCNJ2*. The mutation was a hot spot that caused loss of function and resulted in a dominant-negative effect on Kir2.1 (3). His parents were tested as wild types without mutations (Figure 1B). Therefore, the result showed that our patient carried a *de novo* mutation.

The patient was discharged from our hospital with a prescription of mexiletine (15 mg/kg/day every 8 h). The patient reported no further experience of severe adverse effects. For over 2 years of outpatient follow-up, he remained symptom-free. A repeat 24-h Holter showed < 0.1% PVC burden. The QUc interval was shortened from 680 to 610 ms with no change in U wave amplitude (Supplementary Figure 4).

## Discussion

Andersen-Tawil syndrome (ATS) is a rare inherited disease characterized by specific ventricular arrhythmias including bidirectional or polymorphic ventricular tachycardia (bVT/pVT), periodic paralysis, and dysmorphic features (2). BVT/pVT has been popularly described in ATS excluding digitalis toxicity and catecholaminergic polymorphic ventricular tachycardia (CPVT). Since both ATS and CPVT are rare inherited arrhythmic disorders, there are some clinical similarities between patients with ATS and those with CPVT. Careful differential diagnosis between ATS and CPVT is important before treatment. First, typical QUc prolongation is obvious in widespread limb and/or precordial leads of baseline electrocardiograms in ATS. Second, frequent premature ventricular contractions (PVCs) with right bundle branch block (RBBB) morphology are popular in patients with ATS, while those with left bundle branch block (LBBB) morphology are dominant in patients with CPVT. Third, ventricular arrhythmias are induced during exercise stress test even on effective medication in patients with CPVT while suppressed at peak exercise in patients with ATS (4).

There are two types of ATS based on gene mutation. Type 1 ATS, in which a mutation in the *KCNJ2* gene can be identified, accounts for about 60–70% of all patients with ATS, while type 2 (ATS2), of which the genetic cause is still unknown, accounts for the remaining 30–40% of ATS cases. The *KCNJ2* gene encodes the Kir2.1 inward rectifier potassium channel protein, which mainly causes prolongation of phase 3 of the action potential with reduced inward rectifier potassium current (I_K1_) resulting from *KCNJ2* mutations (3). Cardiac repolarization duration prolongation leads to distinctive T-U wave morphology and a variety of ventricular arrhythmias. According to the latest ATS-iPS cell-derived cardiomyocytes, the mechanism of ventricular arrhythmia is associated with intracellular calcium overload and sodium/calcium exchanger (NCX) mediated triggered activity (5).

In the expert consensus on the treatment of ATS, the β-adrenergic blocker is a primary option including metoprolol or propranolol in China. However, treatment response varies in patients with the type of arrhythmia. Proportional LQT2 patients and a majority of LQT3 patients showed QT interval prolongation in slow heart rate after taking β-blocker. And the efficacy of β-blockers was controversial for counteracting VAs in patients with ATS (6). In our case, the boy experienced apparent fatigue and bradycardia after receiving metoprolol 11.25 mg twice a day for only 2 days. Flecainide is an alternative anti-arrhythmic agent for patients' intolerant of β-blocker. It can significantly suppress ventricular arrhythmia by directly blocking NCX and irregular calcium release besides blocking the fast-inward sodium channel. Additionally, flecainide may activate I_K1_ in ventricular myocytes (7). Unfortunately, flecainide is not yet available in China.

Therefore, the class Ib anti-arrhythmic drug mexiletine was proposed as an alternative therapy for patients with partial LQT2, LQT3, and LQT8 (Timothy Syndrome). To date, mexiletine treatment efficacy in ATS (LQT7) with *KCNJ2* mutations has rarely been reported. The mechanism underlying the suppression of VAs by mexiletine in ATS has not been fully understood. One possible explanation is that mexiletine inhibits the late Na current (I_Na−L_), thereby reducing NCX and intracellular calcium during the repolarization phase. The physiologic I_Na−L_ is intensified by loss of potassium channel function including I_KS_ and I_K1_. Additionally, the increased I_Na−L_ at lower heart rate was named as reverse use-dependence (8), which explains the treatment failure of beta-blocker in patients with ATS. It is important to note that the ratio of the inhibitory concentration at 50% block (IC50) for I_Na−L_ block by mexiletine falls within the therapeutic concentration range (9). There are no studies on the effect of mexiletine on the I_K1_ current. Another interpretation is that mexiletine shortens the action potential duration (APD) of ventricular muscles by activation of the ATP-sensitive potassium channel (K_ATP_) (10), as the QUc interval is shortened by 70 ms in our patient. The most popular adverse effects associated with mexiletine are minor gastrointestinal or neurological effects, which can be tolerated.

In summary, our case demonstrates a new anti-arrhythmic therapy with mexiletine for prevention of life-threatening cardiac events in patients with ATS who are intolerant of β-blocker. Future studies are needed to provide empirical supports for the aforementioned treatment and to further examine the underlying mechanism of its efficacy.

## Data availability statement

The original contributions presented in the study are included in the article/Supplementary materials, further inquiries can be directed to the corresponding author.

## Ethics statement

The studies involving human participants were reviewed and approved by Ethics Committee of Beijing Tsinghua Changgung Hospital. Written informed consent to participate in this study was provided by the participants' legal guardian/next of kin.

## Author contributions

The study was designed by JY and PZ. ECG data collection was performed by KL. The *KCNJ2* gene mutation was identified by TL by Sanger sequencing. The clinical care and treatment follow-up of the patient were performed by YX and FL. All authors contributed to the article, conception of the study, and approved the submitted version.

## Funding

This study was funded by Beijing Municipal Administration of Hospitals Clinical Medicine Development of Special Funding (No. ZYLX201831).

## Conflict of interest

The authors declare that the research was conducted in the absence of any commercial or financial relationships that could be construed as a potential conflict of interest.

## Publisher's note

All claims expressed in this article are solely those of the authors and do not necessarily represent those of their affiliated organizations, or those of the publisher, the editors and the reviewers. Any product that may be evaluated in this article, or claim that may be made by its manufacturer, is not guaranteed or endorsed by the publisher.
