# Supplementary material for: Case report: Mexiletine suppresses ventricular arrhythmias in Andersen-Tawil syndrome
Source: Front Cardiovasc Med. 2022 Aug 25;9:992185. doi: 10.3389/fcvm.2022.992185 (PMC9453449; doi:10.3389/fcvm.2022.992185)
Supplement: Supplementary file 1 [file Data_Sheet_1.pdf]

Supplementary Material

Supplementary Figures

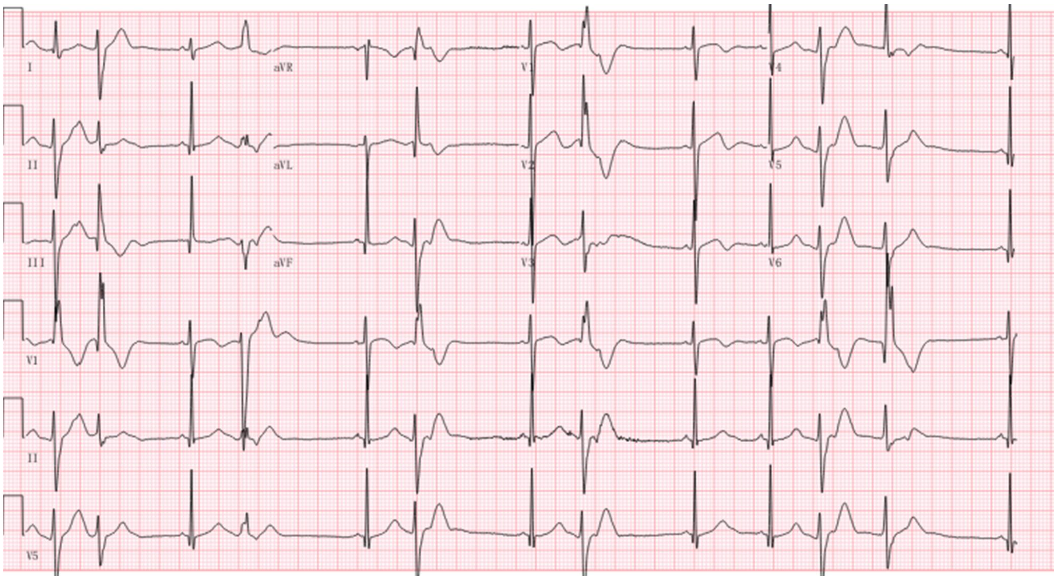

Supplementary Figure 1. The electrocardiogram in our hospital revealed frequent premature ventricular contractions(PVCs) and bidirectional PVCs with QTc of 420 ms and QUc of 680 ms.

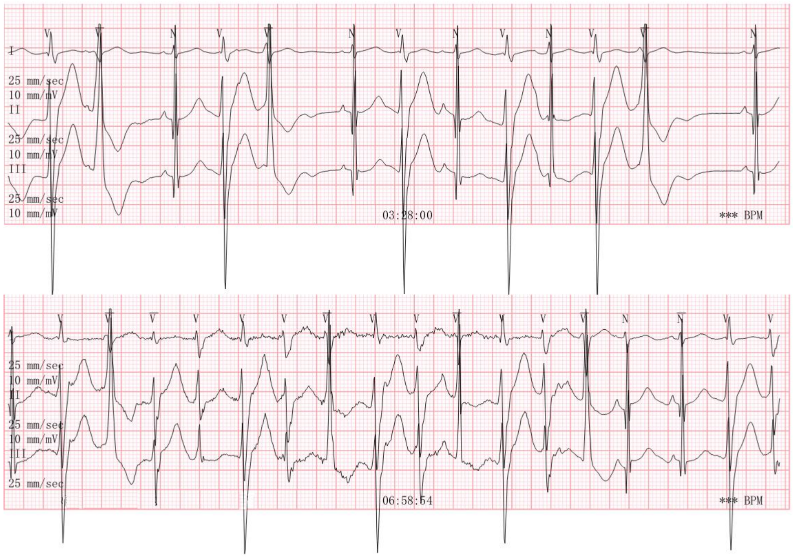

Supplementary Figure 2. Frequency of ventricular arrhythmia in 24-hour Holter recording before taking mexiletine.

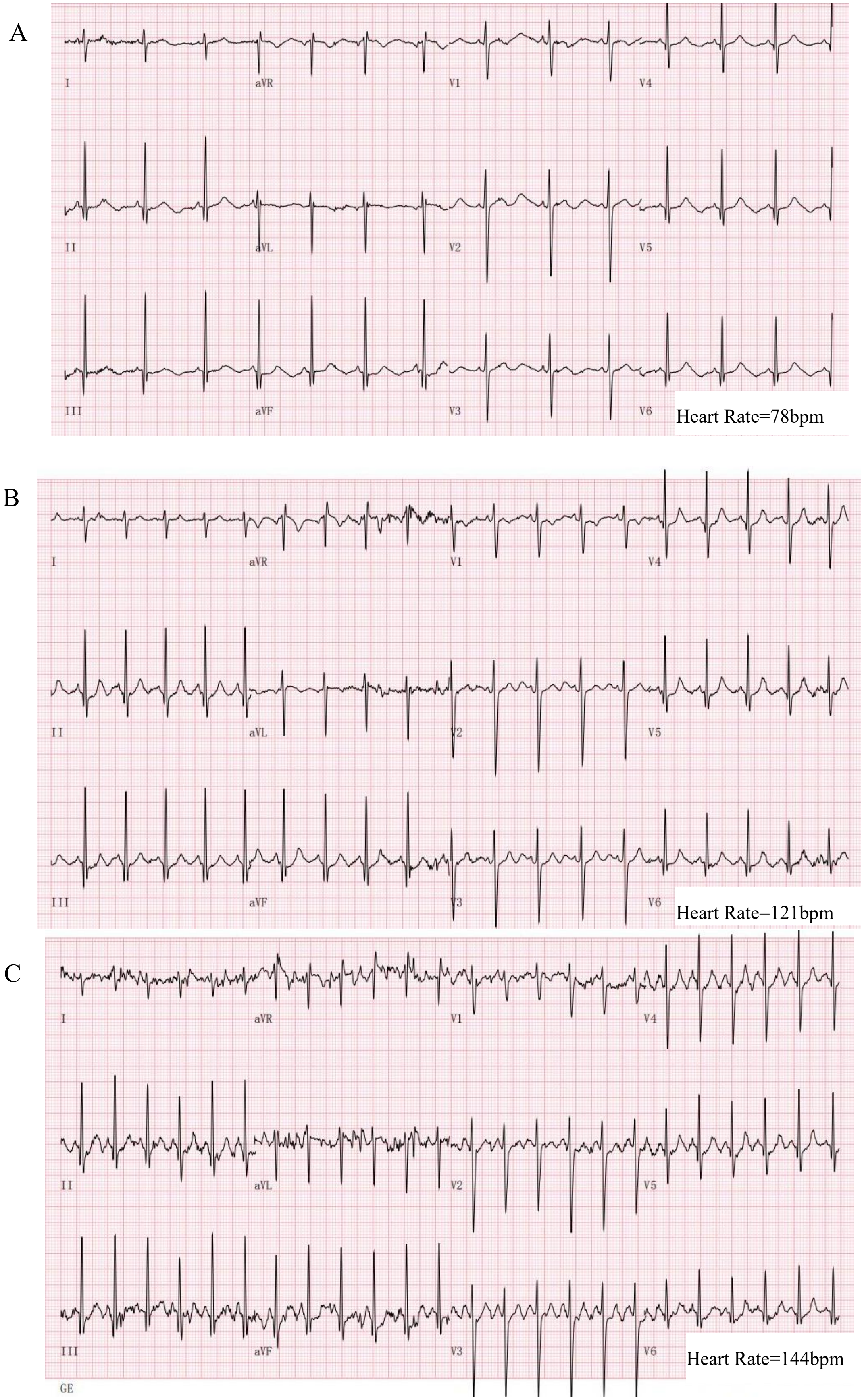
A
I
aVR
V1
V4
II
aVL
V2
V5
III
aVF
V3
V6
Heart Rate=78bpm
B
I
aVR
V1
V4
II
aVL
V2
V5
III
aVF
V3
V6
Heart Rate=121bpm
C
I
aVR
V1
V4
II
aVL
V2
V5
III
aVF
V3
V6
Heart Rate=144bpm
GE

Supplementary Figure 3. Treadmill exercise test revealed QTUc 730ms before test and 590ms after exercise with the heart rate of 121bpm,and U wave infusion with P wave and“U on P” sign (U-wave masquerading P-wave) at peak heart rate 144bpm.

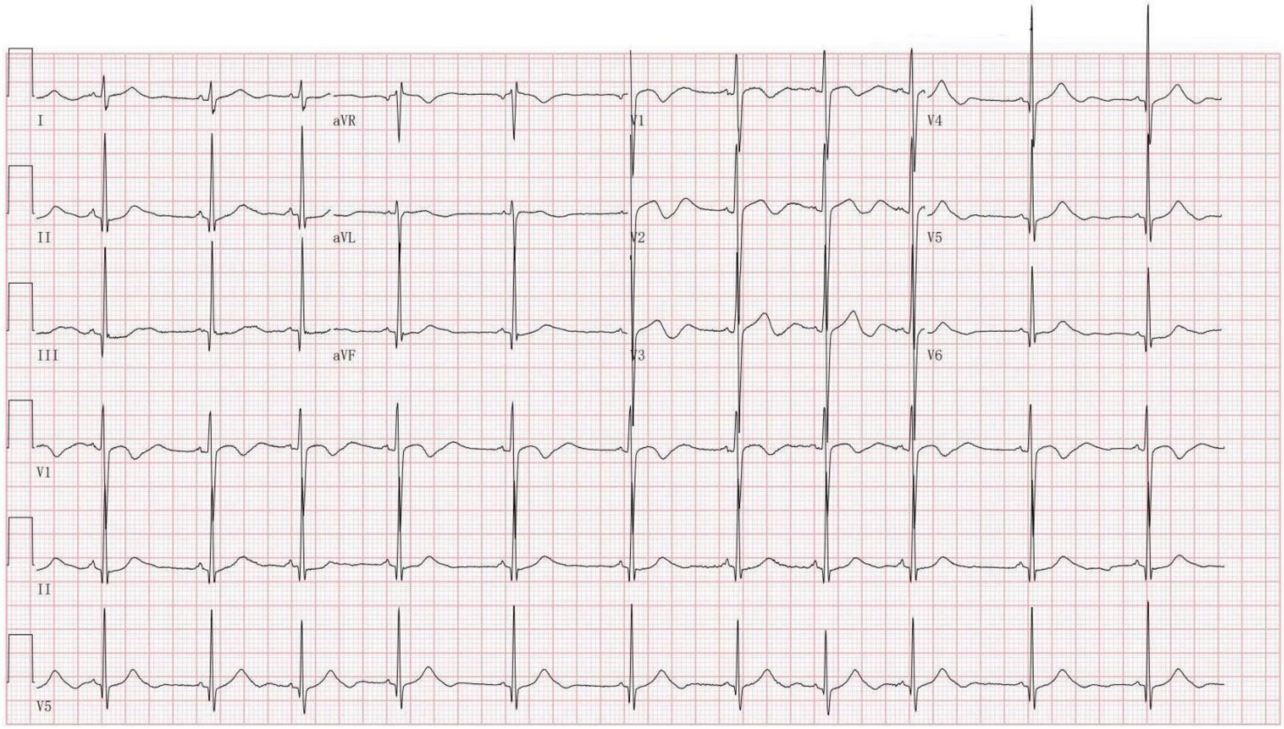

Supplementary Figure 4. The electrocardiogram after taking mexiletine showed the QUc interval was shortened from 680 ms to 610 ms with no change in U wave amplitude.
